# Permeation Characteristics of CH_4_ in PVDF with Crude Oil-Containing

**DOI:** 10.3390/polym14132723

**Published:** 2022-07-03

**Authors:** Xuemin Zhang, Huifang Chu, Houbu Li, Guoquan Qi, Jinmao Feng, Xiong Gao, Wenhui Yang

**Affiliations:** 1 School of Materials Science and Engineering, Chang’an University, Xi’an 710064, China; chuchu123456782021@163.com; 2 WeiXing Group, Zhejiang Weixing New Building Materials Co., Ltd., Linhai 317000, China; zw000809@163.com; 3 State Key Laboratory for Performance and Structure Safety of Petroleum Tubular Goods and Equipment Materials, CNPC Tubular Goods Research Institute, Xi’an 710077, China; lihoubu@cnpc.com.cn (H.L.); qiguoquan@cnpc.com.cn (G.Q.); 4 Shaanxi Yanchang Petroleum Northwest Rubber LLC, Xianyang 712023, China; gaoxiong.69@163.com (X.G.); yunfei000817@163.com (W.Y.)

**Keywords:** thermoplastics, crude oil, solubility, diffusion, gas permeation mechanism, molecular simulation

## Abstract

The liner of reinforced thermoplastic composite pipes (RTPs) used for oil and gas gathering and transportation experienced blister failure due to gas permeation. Few reports have appeared on the problem of gas permeation in thermoplastics with absorbed crude oil. Accordingly, the permeability of CH_4_ in polyvinylidene fluoride (PVDF) containing crude oil was studied at the normal service conditions by molecular simulations. The results showed that the solubility coefficients of CH_4_ in PVDF containing crude oil were much lower than those in pure PVDF. It can be concluded that the crude oil molecules absorbed into PVDF occupied certain adsorption sites, resulting in a decrease in the adsorption capacity of CH_4_ molecules in PVDF. The diffusion coefficients of CH_4_ in oil-containing PVDF were significantly greater than in PVDF. This is because the absorption of oil molecules leads to the volume swelling of PVDF and then increases the free volume for diffusion. The permeation process showed that CH_4_ molecules were selective-aggregate adsorbed in the region with low potential energy in oil-containing PVDF firstly, and then they vibrated within the holes of PVDF containing oil in most cases and jumped into the neighboring holes at high temperatures and pressures.

## 1. Introduction

In the field of oil and gas gathering and transportation, the gas in the transported medium is constituted by methane (CH_4_), carbon dioxide (CO_2_), and hydrogen sulfide (H_2_S) in varying amounts [1], which can corrode the metal pipes used in the process and lead to perforation and other accidents. In recent years, reinforced thermoplastic composite pipes (RTPs) have been widely used instead of conventional carbon steel pipelines in oil and gas gathering and transportation fields due to their excellent corrosion, wear, waxing and scaling resistance, and easy installation [2]. There are three layers in RTPs, including an internal thermoplastic liner, a reinforcement layer, and an outer thermoplastic layer. Among them, the internal thermoplastic liner of RTPs directly contacts the transport medium. However, because of its inherent properties, thermoplastics do not act as perfect barriers. When gases or gas-bearing liquids pass through RTPs, gas molecules permeate into the inner thermoplastics by adsorption, solution, and diffusion phenomena, and consequently accumulate in thermoplastics, resulting in the blister failure of thermoplastics [3]. In the process of oil and gas transportation, the gas-bearing liquid mainly contains crude oil, which can also be absorbed into thermoplastics and then cause their volume expansion, i.e., swelling [4]. Crude oil absorption would inevitably affect the permeation behavior of gases and increase the probability of blister failure of thermoplastics. Therefore, it is necessary to clarify the influence of oil absorption on gas permeability in an oil-gas coupled medium to ensure the safe operation of the pipelines.

At present, much work has been devoted to studying the permeability of gases in polymers by numerous experimental methods such as via the steady state, time lag method, and sorption-desorption tests [5,6,7,8,9,10,11]. Nevertheless, few works have been carried out on the gas permeation in thermoplastics for oil and gas gathering and transportation due to a long experimental process, high cost, and high risk at high pressure. Computational simulation is a useful technique to complement experimental studies, which can not only calculate solubility and diffusion in polymers, but also predict the properties at both the macroscopic and microscopic levels. The existing methods such as the Monte Carlo (MC) [12,13,14] and molecular dynamics (MD) [15,16,17] methods are very desirable to understand and predict the transport properties of gases in polymers. Lu et al. [18] calculated the sorption and diffusion properties of gas molecules through amorphous PPX C polymers by Monte Carlo (MC) and molecular dynamics (MD) simulations. These methods were also performed by Börjesson et al. to calculate solubility and diffusion coefficients of oxygen and water in polyethylene to understand the diffusion mechanism at the molecular level [19]. Eslami and coworkers [20,21] studied the solubility, diffusion, and permeation of gases in polystyrene over a wide range of temperatures by MC and MD simulations, respectively. They found that the calculated solution and diffusion coefficients and the ratios of calculated permeabilities agree well with the experimental data.

Although numerous studies on gas permeability in polymers using experimental and molecular simulations have been done, there is still a lack of understanding on permeation characteristics of gases in oil-gas coupled medium in thermoplastics. Polyvinylidene fluoride (PVDF) is one of the most common thermoplastics used for the liner of RTPs, which is a synthetic resin produced by the polymerization of vinylidene fluoride (CH_2_=CF_2_). It possesses excellent properties such as thermal stability, mechanical strength, aging resistance, and chemical stability [22], and has been widely used in the chemical industry [23], semiconductor manufacturing [24], fluorocarbon coatings [25], membrane science [26,27], oil/water separation [28], and medicine [29]. Usually, the main component of the gases transported in oil and gas gathering and transportation systems is CH_4_, and its content is often more than 95%. Thus, we have performed the permeation behavior and mechanism of CH_4_ molecules in polyvinylidene fluoride (PVDF) at the service conditions in oilfields via molecular simulations [30]. In this work, considering the absorption of crude oil, we focused more specifically on the permeation of CH_4_ molecules in PVDF containing crude oil. The typical crude oil molecules were added in PVDF. The effect of crude oil molecules on CH_4_ permeability characteristics was analyzed under normal service conditions, and the permeation mechanism of CH_4_ in PVDF containing crude oil was discussed.

## 2. Theoretical Basis of Permeation

The solution-diffusion model is a widely recognized permeability mechanism model that can be used to describe the permeation behavior of gas molecules in polymer materials. The permeability of gas is determined by its solubility and diffusion capacity, and the permeability coefficient *P* can be calculated by Equation (1):

(1) P=S×D

where *S* is the solubility coefficient and *D* is the diffusion coefficient.

The solubility coefficient *S* describes the relationship between the concentration *C* and the pressure *P_i_* of the gas in polymers at equilibrium, which can be expressed as Equation (2) [31]:

(2) $$C=K_{D}P_{i}+\frac{C_{H}P_{i}b}{1+P_{i}b}$$

where *K_D_* is the Henry constant; *C_H_* is Langmuir adsorption capacity; *b* is the Langmuir constant; *P_i_* is the pressure of the gas in the polymer (Pa); and *C* is the concentration of gas in the polymer (cm^3^ (STP)·cm^−3^).

The adsorption isotherm method is usually used to solve the solubility coefficient of gas molecules in polymers. The adsorption isotherm can be simulated by the Adsorption Isotherm tool in the Sorption module of Materials Studio to obtain the curves of adsorption gas concentration *C* and the pressure *P_i_*. When *P_i_* is zero, the limit slope of the curves is the solubility coefficient *S*, as shown in Equation (3):

(3) $$S={\underset{p\to 0}{\lim}}_{}\frac{C}{P_{i}}=K_{D}+C_{H}b$$

For diffusion of penetrants in polymers, the center-of-mass mean square displacement (MSD) of the penetrant is shown to pass through three distinct regimes including short-time ballistic regime, anomalous diffusion regime, and Fickian regime [21]. In order to calculate the diffusion coefficients by molecular dynamics simulation, one has to calculate the center-of-mass mean square displacement. For sufficient long times, the MSD becomes linear in time, and the diffusion coefficients can be calculated by Einstein’s equation [32] as follows:

(4) $$D_{}=\frac{1}{6N_{}}{\underset{t\to \infty }{\lim}}_{}\frac{d}{d_{t}}\left(\overset{N}{\underset{i=1}{\sum }}{\left[r_{i}\left(t\right)-r_{i}\left(0\right)\right]}^{2}\right)$$

where *D* is the diffusion coefficient, *N* is the total number of penetrants, *t* is the time, *r*(t) is the centroid position of molecule *i* at time *t*, *r*(0) is its initial position, and [*r_i_*(t)−r*_i_*(0)]^2^ is the MSD of the molecule, denoting the ensemble average.

Since the MSD value has been averaged over *N*, m is taken as the slope of the MSD curve obtained by molecular simulation. The above Equation (4) can be simplified as:

(5) $$D_{}=m/6$$

## 3. Simulation Method

**Figure 1:**
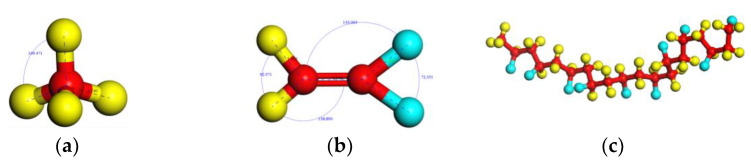
Gas molecules and polymer molecules: (**a**) CH_4_, (**b**) VDF monomer, (**c**) PVDF chain (*n* = 10)_._ Colored balls represent C in red, H in yellow, F in blue.

**Figure 2:**
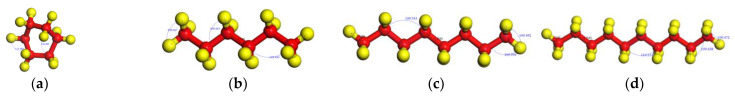
Typical crude oil molecules models: (**a**) C_6_H_12_, (**b**) C_6_H_14_, (**c**) C_8_H_18_, (**d**) C_10_H_22._ Colored balls represent C in red, H in yellow.

The Visualizer module in Materials Studio was used to construct the CH_4_ molecule, vinylidene fluoride (VDF) monomer, and crude oil molecules, respectively, as shown in Figure 1 and Figure 2. Among them, the typical components of crude oil such as hexane (C_6_H_14_), cyclohexane (C_6_H_12_), octane (C_8_H_18_), and decane (C_10_H_22_) [33,34,35] were chosen as oil molecules models, as shown in Figure 2. The PVDF chain was generated with VDF as a repeat unit with a chain length of 10 (Figure 1c). Then, the Smart Minimizer was used to geometry-optimize the above models.

**Figure 3:**
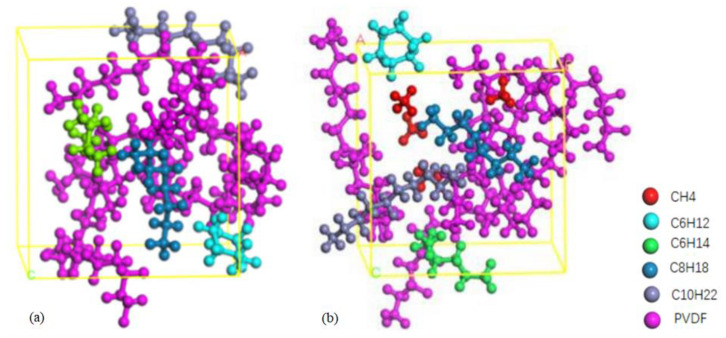
The stable configuration of (**a**) crude oil /PVDF cell, (**b**) CH_4_ /crude oil /PVDF cell.

A three-dimensional cell model of crude oil/PVDF including a hexane, cyclohexane, octane, decane, and four PVDF chains was constructed using the Construct tool in the Amorphous Cell module. After the energy minimization and ensemble optimization, the stable configuration (Figure 3a) can be obtained and used for adsorption simulation. For diffusion simulation, four CH_4_ molecules were added to the crude oil/PVDF cell to construct the CH_4_/crude oil/PVDF mixed-cell model with periodic boundary conditions first. The stable configuration of diffusion (Figure 3b) can be obtained and used for diffusion simulation after the energy minimization and ensemble optimization.

The Grand Canonical Monte Carlo method (GCMC) was adopted, and the Sorption module was applied to calculate the adsorption isotherm and isosteric heats during the sorption process. The adsorption sites were also determined based on the principle of simulated annealing. Additionally, the molecular dynamics method (MD) was adopted and the Forcite module was used to simulate the diffusion process and obtain the atomic trajectories and MSD curve.

Temperatures and pressures were selected for sorption and diffusion simulations according to the actual service conditions of RTPs used in oil and gas gathering and transportation. The temperatures were set at 30 °C, 40 °C, 60 °C, and 80 °C, and the pressures were set at 2.5 MPa, 6.4 MPa, 8.5 MPa, and 10 MPa [36]. Three simulations were conducted at each condition and all the data shown in data graphs were average values. During the simulation, the COMPASS universal force field was employed with high accuracy. The group-based method was used to calculate the van der Waals interaction force, and the Ewald method was used to calculate the electrostatic interaction force. The temperature and pressure were controlled by the Andersen-Berendsen method. The initial velocity of each molecule was randomly distributed via Maxwell-Boltzmann. The simulated step size was 1.0 fs and the total step size was 1000 ps. The trajectory information of the system was recorded every 1000 steps.

## 4. Simulation Results and Discussion

### 4.1. Permeation Behavior of CH_4_ in PVDF Containing Crude Oil

#### 4.1.1. Solubility

**Figure 4:**
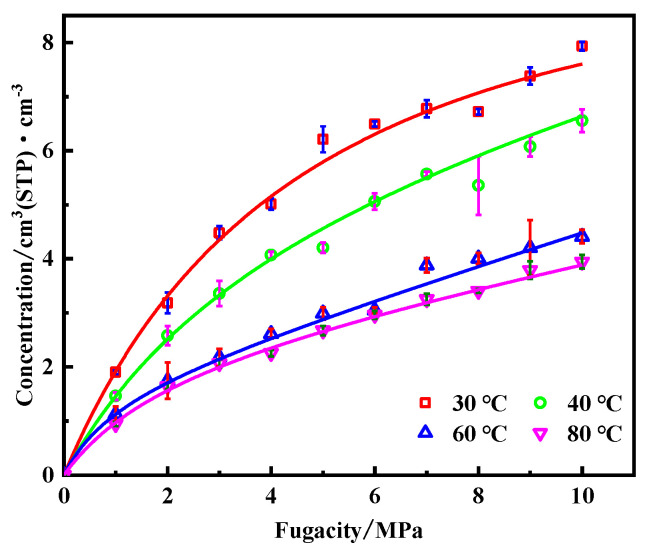
Adsorption isotherm of CH_4_ in PVDF containing crude oil.

**Figure 5:**
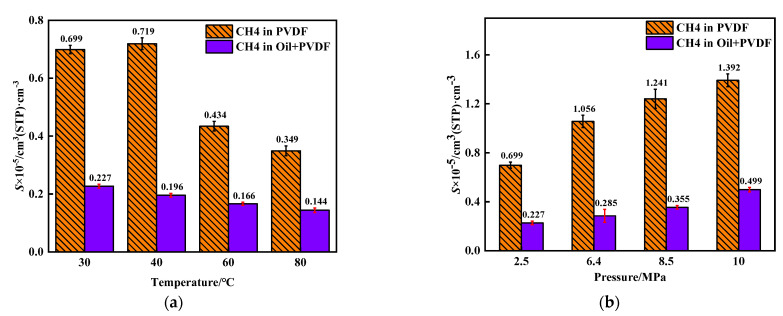
Solubility coefficient of CH_4_ in PVDF at different conditions: (**a**) 2.5 MPa; (**b**) 30 °C.

**Figure 6:**
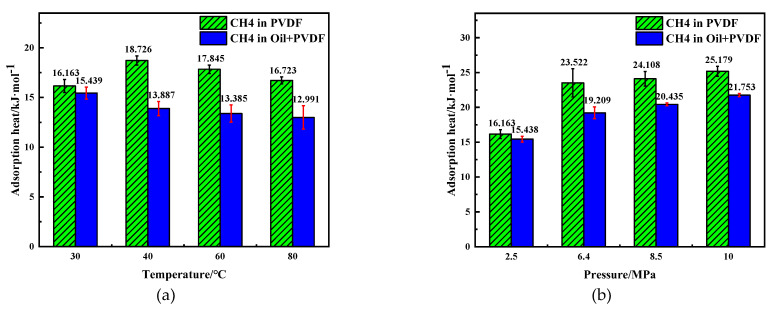
Isosteric heat of adsorption of CH_4_ in PVDF at different conditions: (**a**) 2.5MPa; (**b**) 30 °C.

The adsorption isotherm of CH_4_ molecules in PVDF containing crude oil at different temperatures is shown in Figure 4. The adsorption gas concentration *C* of CH_4_ constantly increases with pressure. The fitting degrees of concentration–fugacity curves with the Langmuir model were 0.9985, 0.9992, 0.9976, and 0.9995, at temperatures of 30–80 °C, respectively. Therefore, the adsorption isotherm of CH_4_ in PVDF containing crude oil corresponds to the Langmuir single-layer reversible adsorption, which is consistent with the previous study on the adsorption of CH_4_ in PVDF [30]. The solubility coefficients of CH_4_ at different conditions can be calculated (Figure 4) according to Equation (3), as shown in Figure 5. The isosteric heat (Q_st_), which is one of the most important indexes to measure the adsorption function of the adsorbent, can reflect the intensity of the adsorption process [37]. The Q_st_ values of adsorption of CH_4_ molecules under different conditions can be obtained from the output file after the simulation, and they are presented in Figure 6. It can be seen that the solubility coefficient (Figure 5) and isosteric heat (Figure 6) decrease with increasing temperature and decreasing pressure. It is well known that the adsorption of gases in polymers is an exothermic process. The isosteric heat decreases with increasing temperature when the adsorption system is at equilibrium. The lower heat indicates weaker interactions between PVDF and CH_4_ molecules, resulting in a lower adsorption amount and a smaller solubility coefficient.

It can also be seen from Figure 5 that the solubility coefficient of CH_4_ in PVDF containing crude oil is much lower than that in pure PVDF. For example, the simulated solubility coefficient for CH_4_ in PVDF ranges from 0.699 × 10^−5^ cm^3^ (STP)·cm^−3^ at 2.5 MPa to 1.399 × 10^−5^ cm^3^ (STP)·cm^−3^ at 10 MPa, while the solubility of CH_4_ in PVDF containing crude oil ranges from 0.227 × 10^−5^ cm^3^ (STP)·cm^−3^ at 2.5 MPa to 0.499 × 10^−5^ cm^3^ (STP)·cm^−3^ at 10 MPa. The largest difference is found at 30 °C and 6.4 MPa, where the solubility of CH_4_ in PVDF containing crude oil is reduced by 73.01%. A similar situation occurred to isosteric heat with a decrease by around 15%, as shown in Figure 6. The crude oil molecules would fill the volume in molecular chains of PVDF after adsorption. When the temperature and pressure increase, the crude oil molecules absorbed into PVDF would occupy certain adsorption sites, resulting in a decrease in the adsorption capacity of CH_4_ molecules in PVDF. Therefore, it can be concluded that the adsorption of crude oil molecules can significantly reduce the solubility of CH_4_ molecules in PVDF.

#### 4.1.2. Diffusion

**Figure 7:**
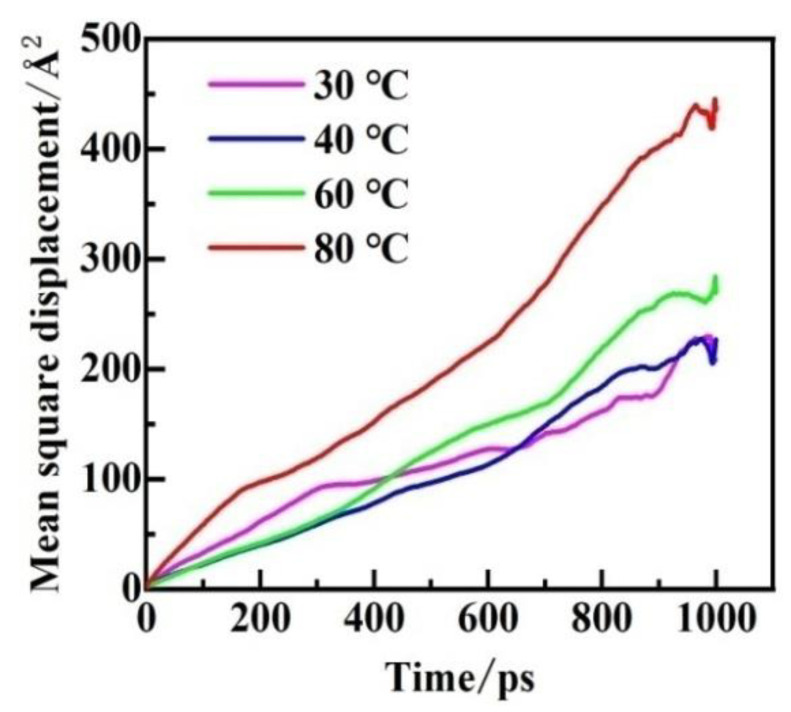
MSD curves of CH_4_ in PVDF containing crude oil at 2.5 MPa.

**Figure 8:**
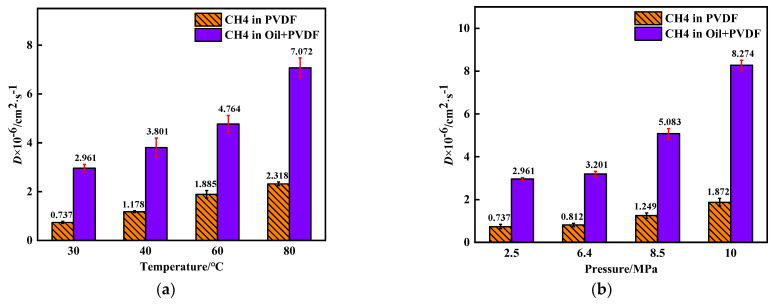
Diffusion coefficient of CH_4_ in PVDF at different conditions: (**a**) 2.5MPa, (**b**) 30 °C.

The MSD of CH_4_ molecules in PVDF containing crude oil with time is shown in Figure 7. The value of MSD is increasing with time, exhibiting a linear relationship. Due to the statistic error, noisy points appear at the end of the curves and need to be eliminated. The diffusion coefficients of CH_4_ were determined by using Equation (5), as shown in Figure 8. As the temperature and pressure increase, the diffusion coefficient increases significantly. Interestingly, the diffusion coefficients of CH_4_ in PVDF containing crude oil are in the range of 2.961 × 10^−6^–8.274 × 10^−6^ cm^2^·s^−1^, which are about three times greater than in pure PVDF under the same conditions. The reason for this phenomenon is that the addition of crude oil molecules may promote gas diffusion.

**Figure 9:**
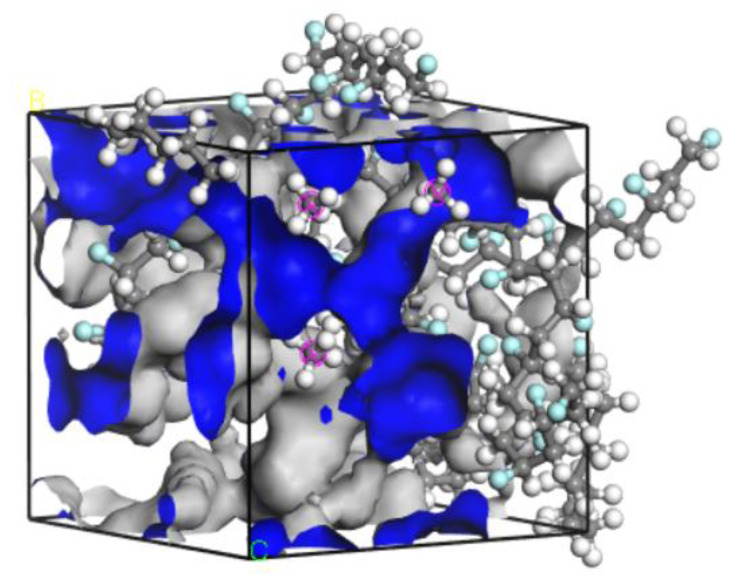
The free volume distribution of CH_4_/(Oil + PVDF) cell.

**Figure 10:**
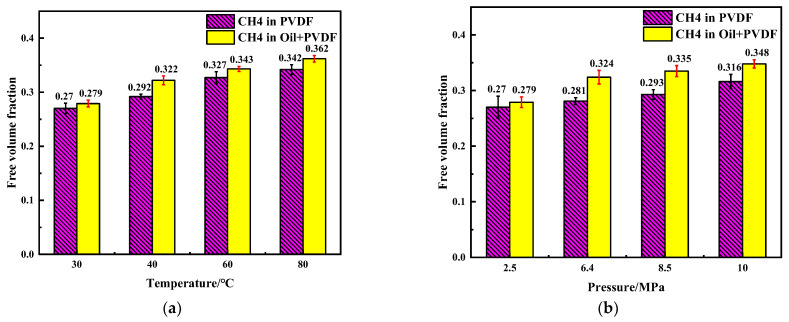
Free volume fraction of CH_4_ in PVDF at different conditions: (**a**) 2.5 MPa, (**b**) 30 °C.

According to free volume theory, the free volume is a measure of the internal space available within a polymer matrix. As the free volume increases, so does the freedom of movement for polymer chains and other small molecules [38,39,40]. The distribution of free volume was simulated using the Atom Volume and Surface module, as the gray area shown in Figure 9. The free volume fraction is the ratio of the free volume and the simulated total cell volume. The fraction values at different temperatures and pressures are shown in Figure 10. Since the stability of the PVDF chain would be reduced at the higher temperatures and pressures, the chain segments are more likely to undergo torsion or relative motion. Then, the free volume would be increased in the simulated cell, and the space for the activity of CH_4_ molecules would as well. As a result, it would be easier for the diffusion process to occur. Moreover, the crude oil molecules absorbed into PVDF play a plasticizing role [30], which can improve the activity of chain segment. The absorption of crude oil molecules is conducive to gas passage and can significantly improve the diffusion ability of CH_4_ molecules in PVDF.

#### 4.1.3. Permeation

**Figure 11:**
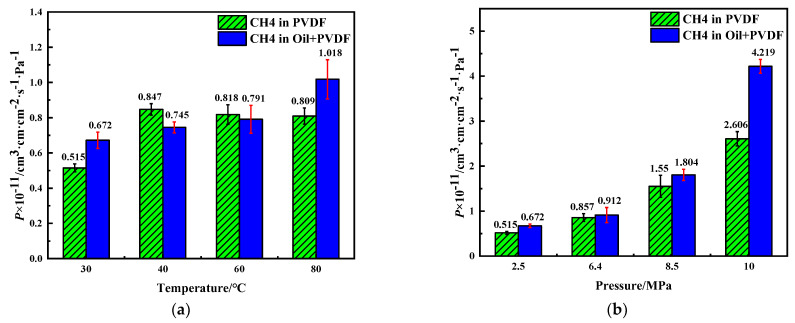
Permeability coefficient of CH_4_ in PVDF under different conditions: (**a**) 2.5 MPa, (**b**) 30 °C.

Based on the solubility coefficient and diffusion coefficient obtained above, the permeability coefficients of CH_4_ in PVDF containing crude oil at different conditions were calculated by Equation (1), as shown in Figure 11. The permeability coefficient of CH_4_ molecules increases in PVDF containing crude oil as the temperature and pressure increase. In addition, the corresponding permeability coefficient is always greater than that in pure PVDF under the same conditions. Therefore, it can be concluded that the absorption of crude oil molecules would in general improve the permeability of gas molecules in PVDF.

### 4.2. Permeation Mechanism of CH_4_ in PVDF Containing Crude Oil

**Figure 12:**
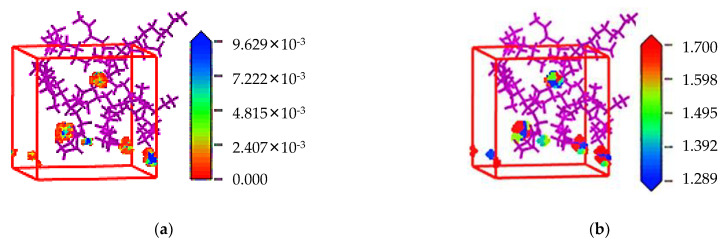
Density field distribution (**a**) and isopycnic (**b**) of CH_4_ in PVDF containing crude oil.

The density field distribution and isopycnic of CH_4_ molecules in PVDF containing crude oil were obtained via the Sorption module to qualitatively study the adsorption sites, as shown in Figure 12. The adsorption density field distribution represents the concentrated area of the density distribution of gas molecules in the simulated cell. The scattered area in Figure 12a is the distribution location of adsorbed CH_4_ molecules in PVDF containing crude oil. It can be seen that the adsorption sites are selective aggregated rather than uniformly distributed. The isopycnic display diagram is a display mode that combines energy and density information. As shown in Figure 12b, the color mapping table on the right side of the figure reflects the energy field value on the isopycnic surface. The smaller the value in the blue region, the lower the intermolecular interaction energy is in this region. According to the principle of minimum energy, gas molecules are most easily adsorbed in the region with the lowest energy, i.e., the blue region in Figure 12b. It can be concluded from Figure 12 that the adsorption sites of CH_4_ molecules in PVDF containing crude oil are located in the low potential energy region of the simulation cell, and the adsorption of gas molecules is selective aggregated. This adsorption process is consistent with the result of CH_4_ molecules in PVDF in the previous study [30], except for the reduction of adsorption sites.

**Figure 13:**
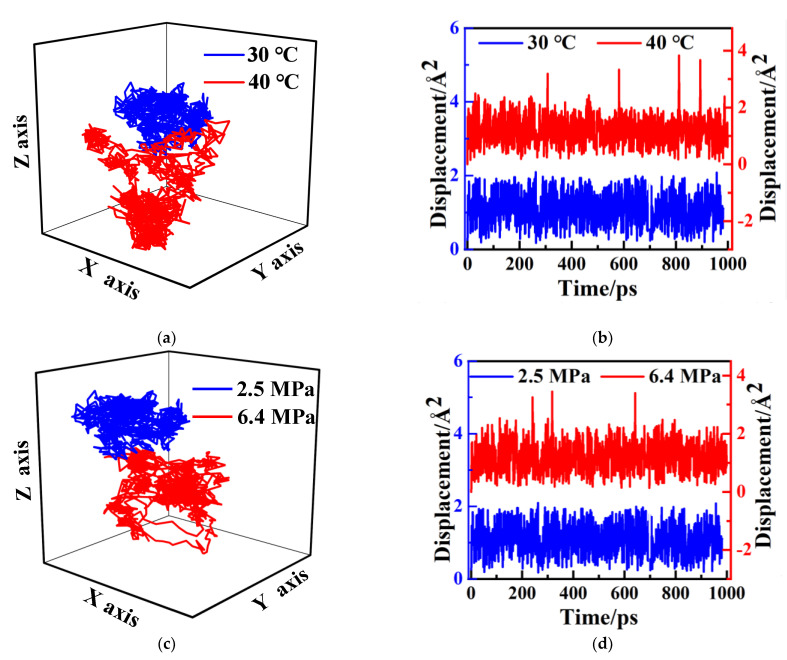
Three-dimensional diffusion trajectory (**a**,**c**) and displacement (**b**,**d**) of CH_4_ molecules in crude oil-containing PVDF at different service conditions.

When the absorbed gas molecules diffuse in the polymer, they vibrate constantly. The molecules not only can diffuse near the vibration position, but also can transmit between different holes, as shown in the previous study [30]. In order to observe the diffusion process of CH_4_ molecules in PVDF containing crude oil, the three-dimensional diffusion trajectory and displacement of CH_4_ molecules at different conditions are shown in Figure 13. It can be seen that CH_4_ molecules basically move back and forth in a certain region (Figure 13a) at the condition of 2.5 MPa and 30 °C, and the amplitudes of the movement displacement are always within 0.1 nm (Figure 13b). When the pressure is constant and the temperature is increased to 40 °C, the diffusion trajectory in space becomes more scattered due to the stronger thermal motion of the CH_4_ molecules (Figure 13a). During the diffusion, there are four large-scale hole-to-hole transitions in the range of 0~1000 ps for CH_4_ molecules. The transition distances are about 0.25 nm (Figure 13b), and the diffusion ability becomes stronger. As the pressure increases from 2.5 MPa to 6.4 MPa, the diffusion trajectory no longer shows the concentrated distribution in the holes (Figure 13c), and there are three large transitions with a transition distance of 0.2 nm (Figure 13d). Therefore, it can be concluded that the diffusion of CH_4_ in PVDF containing crude oil is also combined with the vibration in the holes with the transition among the holes.

Based on the above analysis, the permeation mechanism of CH_4_ in PVDF containing crude oil can be concluded as follows. In PVDF containing crude oil cell, CH_4_ molecules are first selective aggregated in the region with low potential energy, and then they vibrate in the holes. When the temperature or pressure increases, the molecules can jump into the neighboring holes at certain times due to their stronger thermal motion.

## 5. Conclusions

In order to understand the effect of crude oil on the gas permeation in the inner thermoplastic liner of RTPs in oil and gas gathering and transportation systems, Grand Canonical Monte Carlo method (GCMC) and molecular dynamics (MD) simulations were performed to study the molecular-level mechanism as well as the solubility, diffusion, and permeation coefficients of CH_4_ permeating through PVDF containing crude oil. The main results are summarized as follows:

1. Because the crude oil molecules absorbed into PVDF can occupy certain adsorption sites, the solubility coefficients of CH_4_ in PVDF containing crude oil are much lower than in pure PVDF, with drops between 58.74% and 73.01%.
2. The diffusion coefficients of CH_4_ in PVDF containing crude oil are in the range of 2.961 × 10^−6^–8.274 × 10^−6^ cm^2^·s^−1^, which are about three times greater than in pure PVDF under the same conditions. It is considered that the absorption of oil molecules leads to the volume swelling of PVDF and then increases the free volume for diffusion.
3. The absorbed crude oil molecules in PVDF have a significant effect on the permeation process of CH_4_ in PVDF. The absorption of crude oil molecules would improve the permeability of gas molecules in PVDF as a whole.
4. The mechanism of CH_4_ molecules permeated into oil-containing PVDF can be concluded as follows. CH_4_ molecules are selective-aggregation adsorbed in the region with low potential energy in PVDF containing crude oil firstly, then the absorbed CH_4_ molecules vibrate in the holes in most cases, and the calculated movement displacement is always within 0.1 nm. At higher temperatures or pressures, CH_4_ molecules can jump into neighboring holes at certain times due to their stronger thermal motion, and the transition distance is more than 0.2 nm.

## Data Availability

Not applicable.

## References

[1] Sarrasin F., Memari P., Klopffer M., Lachet V., Condat C.T., Rousseau B., Espuche E. (2015). Influence of high pressures on CH4, CO2 and H2S solubility in polyethylene: Experimental and molecular simulation approaches for pure gas and gas mixtures. Modelling of the sorption isotherms. J. Membr. Sci..

[2] Osborne J. (2013). Thermoplastic pipes-lighter, more flexible solutions for oil and gas extraction. Reinf. Plast..

[3] Zhang D., Li H., Qi D., Nan D., Xiaodong S., Bin W., Xuehua C. (2017). Gas Permeation behaviors of high-density polyethylene as a liner material of flexible pipes. Nat. Gas Ind..

[4] Shamsuddoha M., Islam M.M., Aravinthan T., Manalo A., Lau K. (2013). Effectiveness of using fibre-reinforced polymer composites for underwater steel pipeline repairs. Compos. Struct..

[5] Makino Y., Okamoto T., Goto Y., Araki M. The problem of gas permeation in flexible pipe. Proceedings of the Offshore Technology Conference (OTC-5745-MS).

[6] Teplyakov V., Meares P. (1990). Correlation aspects of the selective gas permeabilities of polymeric materials and membranes. Gas Sep. Purif..

[7] Mahallati P., Arefazar A., Naderi G. (2011). Thermal and morphological properties of thermoplastic elastomer nanocomposites based on PA6/NBR. Iran. J. Chem. Eng..

[8] Katoch S., Sharma V., Kundu P.P. (2011). Swelling kinetics of unsaturated polyester and their montmorillonite filled nanocomposite synthesized from glycolyzed PET. Diffus. Fundam..

[9] Minelli M., Sarti G.C. (2013). Permeability and diffusivity of CO_2_ in glassy polymers with and without plasticization. J. Membr. Sci..

[10] Li H., Freeman B.D., Ekiner O.M. (2011). Gas permeation properties of Poly(urethane-urea) containing different polyethers. J. Membr. Sci..

[11] Georage S.C., Thomas S. (2011). Transport phenomena through polymeric systems. Prog. Polym. Sci..

[12] Yang Y., Nair A.K.N., Sun S. (2019). Adsorption and diffusion of methane and carbon dioxide in amorphous regions of cross-linked polyethylene: A molecular simulation study. Ind. Eng. Chem. Res..

[13] Dubbeldam D., Calero S., Ellis D.E., Snurr R.Q. (2016). Molecular simulation software for adsorption and diffusion in flexible nanoporous materials. Mol. Simul..

[14] Kadoura A., Nair A.K.N. (2017). Molecular simulation study of montmorillonite in contact with variably wet supercritical carbon dioxide. J. Phys. Chem. C.

[15] Hui W., Yong X. (2017). Molecular dynamics simulation of gas diffusion behavior in polyethylene terephthalate/aluminium/polyethylene interface. Compos. Interfaces.

[16] Raptis T.E., Raptis V.E., Samios J. (2012). Quantitative study of diffusion jumps in atomistic simulations of model gas–polymer systems. Mol. Phys..

[17] Dutta R.C., Bhatia S.K. (2017). Transport diffusion of light gases in Polyethylene using atomistic simulations. Langmuir.

[18] Lu C., Ni S., Chen W.-K., Liao J., Zhang C. (2010). A molecular modeling study on small molecule gas transportation in poly (chloro-p-xylylene). Comput. Mater. Sci..

[19] Börjesson A., Erdtman E., Ahlström P., Berlin M., Andersson T., Bolton K. (2013). Molecular modelling of oxygen and water permeation in polyethylene. Polymer.

[20] Eslami H., Müller-Plathe F. (2007). Molecular dynamics simulation of sorption of gases in polystyrene. Macromolecules.

[21] Mozaffari F., Eslami H., Moghadasi J. (2010). Molecular dynamics simulation of diffusion and permeation of gases in polystyrene. Polymer.

[22] van Goethem C., de Beeck D.O., Ilyas A., Thijs M., Aerts P., Vankelocom I. (2021). Ultra-thin and highly porous PVDF-filters prepared via phase inversion for potential medical (COVID-19) and industrial use. J. Membr. Sci..

[23] Chen B., Qin S., Zhang X. (2019). Preparation technology of high voltage thin film based on PVDF. Sens. Microsyst. J. Cent. South Univ..

[24] Kar G.P., Biswas S., Bose S. (2016). X-ray micro computed tomography, segmental relaxation and crystallization kinetics in interfacial stabilized co-continuous immiscible PVDF/ABS blends. Polymer.

[25] Governal R.A. (1994). Ultrapure Water: A battle every step of the way. Semicond. Int..

[26] Kim H., Lee S., Shin Y.R., Choi Y.-N., Yoon J., Ryu M., Lee J.W., Lee H. (2022). Durable superhydrophobic poly(vinylidene fluoride) (PVDF)-based nanofibrous membranes for reusable air filters. Appl. Polym. Mater..

[27] Fei F., Le Phuong H.A., Blanford C.F., Szekely G. (2019). Tailoring the Performance of Organic Solvent Nanofiltration Membranes with Biophenol Coatings. Appl. Polym. Mater..

[28] Gao J., Wang J., Xu Q., Wu S., Chen Y. (2021). Regenerated cellulose strongly adhered by a supramolecular adhesive onto the PVDF membrane for a highly efficient oil/water separation. Green Chem..

[29] Mansha M., Salhi B., Ali S., Khan S., Baig N. (2022). Novel procaine-based gemini zwitterion incorporated PVDF membranes for efficient treatment of oily waste water. J. Environ. Chem. Eng..

[30] Zhang X., Wang P., Li H., Chu H., Ding H., Gao X. (2021). Molecular simulation of permeation behavior and mechanism of CH_4_ in PVDF. China Plast..

[31] Theodorou D.N., Suter U.W. (1986). Atomistic modeling of mechanical properties of polymeric glasses. Macromolecules.

[32] Boyd R.H., Pant P.V.K. (1991). Molecular packing and diffusion in polyisobutylene. Macromolecules.

[33] Sakher F., Imqum A. (2020). An experimental investigation of immiscible carbon dioxide interactions with crude oil: Oil swelling and asphaltene agitation. Fuel.

[34] Yang C., Gu Y. (2006). Diffusion coefficients and oil swelling factors of carbon dioxide, methane, ethane, propane, and their Mixtures in heavy oil. Fluid Phase Equilibria.

[35] Zhang R., Mattice W.L. (1993). Flexibility of a new thermoplastic polyimide studied with molecular simulations. Macromolecules.

[36] (2020). Non-Metallic Composite pipe for Petroleum and Natural gas Industries—Part 2: Flexible Composite Pipe for High Pressure Transmission.

[37] Laney P. (2002). Use of composite pipe materials in the transportation of natural gas. INEEL Field Work Proposal..

[38] Langer E., Bortel K., Waskiewicz S. (2019). Plasticizers Derived from Post-Consumer PET.

[39] Trohalaki S., Kloczkowski A., Mark J.E. (1991). Estimation of diffusion coefficient for small molecular penetrants in amorphous polyethylene. Comput. Simul. Polym..

[40] Fried J.R., Sadat-Akhavi M., Mark J.E. (1998). Molecular simulation of gas permeability: Poly (2,6-dimethyl-1,4-phenylene oxide). J. Membr. Sci..

